# The Evolution of Chromosome Numbers: Mechanistic Models and Experimental Approaches

**DOI:** 10.1093/gbe/evaa220

**Published:** 2020-10-14

**Authors:** Itay Mayrose, Martin A Lysak

**Affiliations:** 1 School of Plant Sciences and Food Security, George S. Wise Faculty of Life Sciences, Tel Aviv University, Israel; 2 CEITEC—Central European Institute of Technology, Masaryk University, Brno, Czech Republic

**Keywords:** chromosome numbers, polyploidy, dysploidy, cytogenomics, phylogenetic models, genome evolution

## Abstract

Chromosome numbers have been widely used to describe the most fundamental genomic attribute of an organism or a lineage. Although providing strong phylogenetic signal, chromosome numbers vary remarkably among eukaryotes at all levels of taxonomic resolution. Changes in chromosome numbers regularly serve as indication of major genomic events, most notably polyploidy and dysploidy. Here, we review recent advancements in our ability to make inferences regarding historical events that led to alterations in the number of chromosomes of a lineage. We first describe the mechanistic processes underlying changes in chromosome numbers, focusing on structural chromosomal rearrangements. Then, we focus on experimental procedures, encompassing comparative cytogenomics and genomics approaches, and on computational methodologies that are based on explicit models of chromosome-number evolution. Together, these tools offer valuable predictions regarding historical events that have changed chromosome numbers and genome structures, as well as their phylogenetic and temporal placements.

**Significance**

Chromosome numbers have been widely used to describe the karyotype of eukaryote genomes. Changes in chromosome numbers regularly serve as indication of major genomic events, most notably polyploidy and dysploidy. Here, we describe mechanistic processes underlying changes in chromosome numbers and review recent advancements in the field that increase our ability to make inferences regarding historical events that led to alterations in the number of chromosomes of a lineage.

## Chromosome Number Variation in Land Plants

The chromosome number of a taxon is perhaps the single most informative character that describes the genomic organization of a lineage. For over a century, chromosome numbers have been used as informative phylogenetic indicators (Guerra 2008) and their importance in macroevolutionary processes has been repeatedly discussed (Mayr 1982; Clark and Donoghue 2018). Although considerable variation exists in animals, the extent of this variation has been particularly well appreciated in land plants, inspiring botanists to inspect and document chromosome numbers of many thousands of species (Rice et al. 2015). The lowest number of chromosomes of a plant genome was reported for six angiosperm species that are known to possess merely two chromosome pairs (*n *=* *2; Castiglione and Cremonini 2012). At the other end, extremely high chromosome numbers were reported in the fern *Ophioglossum reticulatum* (*n *=* *720; Khandelwal 1990), the dicot tree *Strasburgeria robusta* (*n *=* *250; Oginuma et al. 2006), the succulent *Sedum suaveolens* (*n* =∼320; Uhl 1978), and the forest coconut palm (*Voanioala gerardii*, *n *=* *303; Röser 2015).

The extant chromosome-number variation is external manifestation of the underlying dynamic genomic processes, encompassing structural chromosomal rearrangements and changes in the DNA content. The most recognizable chromosome-number change is through a whole-genome duplication (WGD), or more generally polyploidization, which describes the acquisition of one or more complete chromosome sets to the genome. Single-chromosome changes represent another common pathway underlying chromosome-number variation. These transitions include the gain/loss of a single chromosome(s)—a process known as aneuploidy, and processes such as chromosome fission and fusion (ascending and descending dysploidy, respectively), which change the chromosome number while preserving most of the genomic content.

The size and morphology of chromosomes change through double-strand breaks (DSBs) in chromosomal DNA and by subsequent miss-repair at these breakpoints. Duplications, deletions, inversions, and translocations, and sometimes combination of these rearrangements, have the potential to alter the length of chromosome arms, change the centromere position, as well as the order and position of genes on chromosomes (gene linkage). Chromosome translocations mediate the reduction of chromosome numbers through recombination between at least two nonhomologous chromosomes (descending dysploidy). Conversely, chromosome breakage not followed by DSB (miss-)repair can potentially result in chromosome-number increase (ascending dysploidy).

During the last decade, revolutionary advancements have enhanced our ability to make inferences regarding historical events that led to chromosome-number changes. These include both experimental procedures, encompassing novel comparative genomics approaches, and computational methodologies that offer more robust and flexible predictions of ancestral chromosome numbers and their phylogenetic placements. Here, we first describe mechanistic processes underlying changes in chromosome numbers. Then, we focus on state-of-the-art experimental and computational methodologies that are applied to uncover such changes and to estimate their timings.

## Processes Governing Chromosome-Number Variation

### Mechanisms of Chromosome Number Increase

#### Polyploidy

Mechanisms underlying polyploidization events have been thoroughly discussed in the literature and so we only briefly describe these here and refer the readers to many excellent reviews (Ramsey and Schemske 1998, 2002; Comai 2005; Otto 2007; Soltis et al. 2016; Van De Peer et al. 2017). Chromosome number sets can be multiplied through somatic doubling, polyspermy, or most frequently, through unreduced gametes that contain the same number of chromosomes as somatic cells. Two types of polyploidy, differentiated by the extent of similarity between the duplicated chromosome sets, have been widely recognized. Autopolyploidy is referred to the condition where the duplicated genomes are nearly identical, having originated from a single species, whereas allopolyploidy is the result of a hybridization of two different lineages (typically species) whose genomes have already diverged to some extent. In case of these euploid changes, the least increment of chromosome number is triploidy (2*n* + *n*), typically produced through fusion of reduced and unreduced gametes or by crossing diploids and tetraploids. Although triploidy is an unstable evolutionary condition, usually characterized by low fertility, it may serve as a route to tetraploidy via the triploid bridge pathway. Tetraploids may originate from the union of *2n* gametes in the triploid via self-fertilization or by backcrossing of the triploid with a closely related diploid individual (Ramsey and Schemske 1998). Tetraploids can also be formed via the fusion of two unreduced gametes, whose frequency is particularly high in hybrids (27.5% compared with 0.56% in nonhybrids). From these, higher ploidy individuals and populations may be generated via a variety of mechanisms, including production of unreduced gametes in polyploid individuals and hybridizations involving one or more polyploid lineages (Ramsey and Schemske 1998).

#### Aneuploidy

Aside from euploid changes, chromosome numbers may increase in a step-wise manner by one or few chromosomes, a process generally referred to as aneuploidy. Aneuploidy refers to extra homologous chromosome(s) being present within a chromosome set during a life span of their carriers or in one or a few subsequent generations (e.g., trisomy of a single chromosome leading to a 2*n *+* *1 genome). Aneuploids may originate by several ways, with nondisjunction in meiosis or mitosis being the most frequent pathway. Meiotic nondisjunction leads to formation of aneuploid gametes. The subsequent union of aneuploid and euploid gamete may result in the origin of trisomic [*n *+ (*n* + 1) → 2*n* + 1] and monosomic [*n* + (*n* − 1) → 2*n* − 1] individuals. In plants, products of mitotic nondisjunction can enter the germline and become transmitted to the progeny due to pluripotency of plant cells.

Frequently, the extra chromosome(s) and/or their carriers are eliminated from the population. However, the union of two *n* + 1 gametes may increase the chromosome number and restore the euploidy in the offspring (2*n* + 2). The resulting tetrasomic progeny will suffer from reduced fertility due to four homologs forming a quadrivalent, followed by irregular chromosome segregation. These individuals are expected to suffer from additional fitness disadvantage due to imbalanced gene content (Otto 2007). Accumulation of structural and DNA changes among the four homologs will eventually restore regular (bivalent) pairing. Still, long-term establishment of such lineages is an exceedingly rare event.

#### Ascending Dysploidy

Mutations causing the increase of chromosome numbers while preserving the genomic content are referred to as ascending dysploidy. Centric fission is traditionally thought to be the most common type of ascending dysploidy. The breakage within a functional centromere or centromere miss-division during chromosome segregation (Birchler and Han 2018) produces two telocentric chromosomes. For this mutation to be stable, that is, an inheritable chromosome-number increase by one (*n* + 1), the compromised centromeres should retain the capacity to form a kinetochore and the centric ends of the two telocentric chromosomes should be healed by de novo telomere formation (Jankowska et al. 2015; Kurzhals et al. 2017). Although ascending dysploidy mediated by centric fissions is assumed to be frequent only in a few plant groups with monocentric chromosomes (e.g., in cycad genus *Zamia*; Rastogi and Ohri 2020), land plant genome evolution is dominated by descending dysploidy (Carta et al. 2020).

### Mechanisms of Chromosome Number Decrease (Descending Dysploidy)

Mitotic or meiotic nondisjunction will render some cells aneuploid (e.g., 2*n*−1; a monosomy of one chromosome). Such aneuploidies are usually not tolerated due to the loss of essential genes. However, in rare circumstances, gene redundancy of polyploid genomes may allow the accidental droppage of some chromosomes without fatal consequences for carrier’s fertility (Clausen and Cameron 1944).

**Fig. 1:**
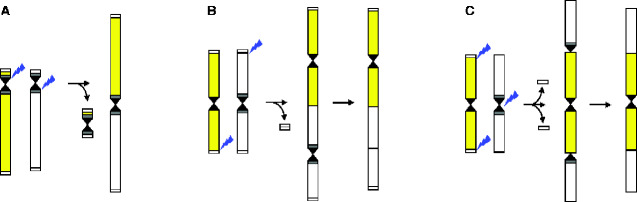
Mechanisms of descending dysploidy in plants. (*A*) Robertsonian translocation. (*B*) End-to-end translocation. (*C*) Nested chromosome insertion. The blue lightning symbols denote the location of double-strand breaks (breakpoints), the black sandglass symbols represent centromeres, and the small white/gray rectangles stand for (sub)telomeric/pericentromeric repeats.

A more common reduction in chromosome numbers, referred to as descending dysploidy, occurs via chromosome fusion. The basis of all descending dysploidies is the mis-repair of DSBs on two or more nonhomologous chromosomes, that is, a chromosome translocation(s). The so-called translocation or “fusion” chromosomes can be transmitted to the offspring and become fixed only at the condition that no housekeeping, or other essential genes, are lost during the process. In organisms with monocentric chromosomes (i.e., with a localized centromere), descending dysploidy is usually accompanied by elimination of one of the two centromeres of the ancestral chromosomes. However, if two nonhomologous centromeres of a fusion chromosome are at a short physical distance, both centromeres may retain their functionality (Page and Shaffer 1998). If more than two chromosomes have contributed to the origin of a fusion chromosome, a more parsimonious step-wise reconstruction, involving the merging of two chromosomes at a time, is usually assumed. However, it is well plausible that in some instances concurrent DSBs on three or more nonhomologous chromosomes would result in structurally complex fusion chromosomes (e.g., Zhang et al. 2008; Mandáková et al. 2019; reviewed by Pellestor 2019). With the advent of high-throughput sequencing technologies and modern cytogenomics, several mechanisms of descending dysploidy were characterized in greater detail or newly identified. The most common types of chromosome-number-reducing mechanisms are illustrated in figure 1 and are briefly described below.

*Robertsonian translocations (ROB; fig. 1A)*. A typical ROB combines long arms of two telo- or acrocentric chromosomes by recombination between their centric ends or short arms. The first translocation product is the fusion chromosome containing two long arms and one or two centromeres (monocentric or dicentric ROB). The second product is either an acentric fragment or centromere-containing mini-chromosome, whose elimination due to its small size and absence of essential genes can be tolerated by the translocation carrier. Thus, ROBs reduce the number of short arms, while increasing the number of metacentric chromosomes, and hence the karyotype symmetry.

*End-to-end translocation (EET; fig. 1B)*. EETs were deduced from two ancestral chromosomes being tandemly arranged within a single evolutionary younger chromosome. As the head-to-tail collinearity of both ancestral chromosomes remains conserved within the fusion chromosome, recombination between uncapped terminal sequences of two nonhomologous chromosomes is the most plausible pathway of these chromosome fusions. Except for instances where the two centromeres are in a tight spatial proximity (i.e., in case of telocentric chromosomes), the fixation of the fusion chromosome depends on elimination of one of the ancestral centromeres. The elimination process is not well characterized and epigenetic modifications together with recombination-dependent deletion of centromere-specific nucleosomes and DNA sequences are purported to instantly restore monocentricity of the fusion chromosome (Lysak 2014).

*Nested chromosome insertion (NCI; fig. 1C)*. NCIs combine two ancestral nonhomologous chromosomes in a peculiar fashion, superficially appearing as an insertion of one chromosome into the (peri)centromere of another chromosome (Luo et al. 2009). The “recipient” chromosome undergoes a centric fission followed by recombination between its two centric ends and sequences on both ends of the “insertion” chromosome; providing that both ends of the insertion chromosome lose their protective telomere structures. Thus, the centromere of the insertion chromosome serves as the functional centromere, whereas the centromere of the recipient chromosome loses its function.

#### Dysploidy in Groups with Holocentric Chromosomes

In organisms with monocentric chromosomes, all chromosomal rearrangements increasing or reducing the number of chromosomes must comply with the persistence or elimination of a functional centromere. In contrast, in organisms with holocentric or holokinetic chromosomes, that is, chromosomes with a kinetochore assembling along the entire chromosome length, dysploid changes are more easily fixed. The frequent fission and fusion events acting on holocentric chromosomes result in long dysploid series of chromosome numbers in some plant genera, such as *Carex* and *Luzula* (Guerra 2016 and references therein), as well as in butterflies (Lepidoptera; e.g., Ahola et al. 2014).

## Methodological Approaches to Analyze Chromosome-Number Variation and Chromosome Collinearity

As all inferences of chromosome-number evolution are based on extant chromosome-number variation, all predictions essentially start on a laboratory workbench by establishing chromosome numbers for species in a group of interest. However, chromosome number itself usually does not provide sufficient information on how the individual chromosomes and the whole karyotypes originated, and how they are related to chromosome complements of other species. Hence, the chromosome structure and cross-species chromosome/genome collinearity are being examined by comparative cytogenomic and genomic approaches.

### Chromosome Counts and Ploidy Estimation

Chromosome counting from floral (e.g., anthers, pistills) and vegetative (most frequently root tip meristems) plant tissues still remains the most reliable method to establish the chromosome number of an investigated individual. This is often a laborious, time-consuming, and occasionally unsatisfactory procedure (e.g., in polyploid species with high chromosome numbers) that cannot be applied to large population samples. Although flow-cytometric DNA content estimation cannot substitute chromosome counting (Suda et al. 2006), it may represent a practical variant of deducing chromosome numbers and ploidy levels, unless genome size in the examined clade varies considerably.

### Comparative Cytogenomic Approaches

Although chromosome counting and ploidy estimation methods provide the information on the number of chromosomes, chromosome structure and rearrangements underlying chromosome number variation have to be determined through comparative analysis of individual chromosomal DNA molecules. Direct localization of DNA sequences on chromosomes provides an observable evidence of their physical position. The development of cytogenetic methods used in comparative plant genomics was driven by three principal requirements. First, DNA sequences (called DNA probes) should be chromosome-specific, with none or minimal off-target localization. Second, a DNA probe should identify long stretches of chromosomal DNA (i.e., from hundreds of kb- to Mb-long regions, up to entire chromosomes). Third, comparative analyses require cross-hybridization of chromosome-specific DNA probes among two or more genomes being compared. This means that intergenome sequence homeology must be sufficiently high to ensure that a probe originating from one genome will target a homeologous chromosomal region in other genomes. Thus, the level of phylogenetic relatedness among the compared genomes is a critical parameter.

With the advent of DNA sequencing and DNA cloning technologies, methods of classical comparative cytogenetics have changed (for a recent review, see Hu et al. 2020), enabling researchers to target and compare DNA sequences of individual chromosomes. This conceptual shift transformed classical cytogenetics research into comparative cytogenomics—the discipline of chromosome research capitalizing on multiple whole-genome sequences. Some of these approaches are detailed below.

### Comparative Cytogenomics

#### Comparative Chromosome Painting Based on Bacterial Artificial Chromosomes

**Fig. 2:**
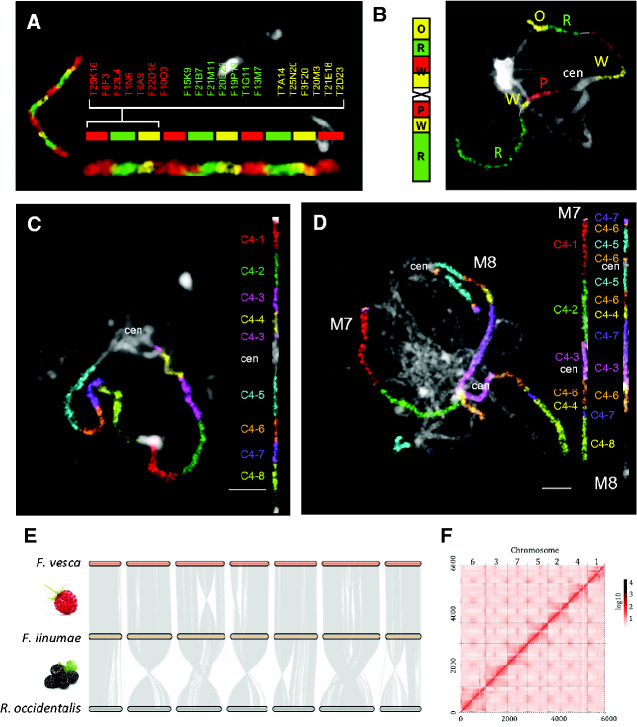
Complementary approaches of comparative plant genomics. (*A* and *B*) BAC-based chromosome painting (CP). (*A*) CP using chromosome-1-specific BAC clones of *A. thaliana* on pachytene chromosomes of this species. The set of 66 BACs (∼6.7 Mb) was arbitrarily divided into ten alternatively labeled BAC contigs. (*B*) Comparative BAC-based CP. Pachytene chromosome 6 of *Noccaea caerulescens* (Alpine Penny-cress) painted using chromosome-specific BAC clones of *A. thaliana*. Capital letters refer to ancestral genomic blocks. cen, centromere. (*C* and *D*) Oligo painting. (*C*) Multiplex PCR-based oligo painting (MP-OP) using eight oligo probes specific for cucumber (*Cucumis sativus*) chromosome 4 on pachytene chromosomes of this species. (*D*) Comparative MP-OP using the same probes as in (C) revealing two homeologous chromosomes (M7 and M8) in the melon genome (*Cucumis melo*). (*E*) Chromosome-scale genome comparison among two strawberry (*Fragaria*) and one black raspberry (*Rubus*) species revealing the conserved versus corrupted intergenome chromosome collinearity. All three genomes were sequenced and assembled using the PacBio single-molecule sequencing technique (Edger et al. 2018, 2020; VanBuren et al. 2018). (*F*) High-throughput chromosome conformation capture (Hi-C) map of the *Rubus occidentalis* genome. Putative locations of centromeres are visible for some of the seven chromosomes. Figures were contributed by the authors of Mandáková and Lysak 2016 (*A*), Mandáková et al. 2015 (*B*), Bi et al. (2020) (*C* and *D*), Hardigan et al. (2020) (*E*), and VanBuren et al. (2018) (*F*).

Bacterial artificial chromosomes (BACs) are large-insert vectors containing fragments of chromosomal DNA (∼100 kb or larger). A set of BAC clones covering an entire chromosome or chromosome arm is referred to as a chromosome-specific library, whereas a continuous array of overlapping DNA clones is called a BAC contig (a BAC tiling path). Initial attempts to localize individual chromosome-specific BACs and short BAC contigs on plant chromosomes using fluorescence in situ hybridization (FISH) date to the mid-1990s (Woo et al. 1994; Hanson et al. 1995; Jiang et al. 1995), and were followed by numerous studies applying BAC FISH (reviewed by Jiang and Gill 2006; Jiang 2019). Large-scale chromosome painting to identify entire chromosomes using BAC contigs was established only for crucifers (Brassicaceae) and grasses (Poaceae). Lysak et al. (2001) published a proof-of-concept study showing BAC painting of the shortest *Arabidopsis thaliana* chromosome using 139 clones covering almost 16 Mb of the chromosome. Later, Pecinka et al. (2004) were able to BAC paint all five Arabidopsis chromosomes along their entire length. The true power of BAC-based chromosome painting for plant comparative genomics was demonstrated by application of Arabidopsis chromosome-level BAC contigs to analyze karyotypes of other crucifer species (fig. 2*A* and *B*) (Lysak et al. 2005, 2006). This approach, exploiting low- and single-copy (orthologous) sequences ancestrally shared among genomes, allows for identification of homeologous chromosome regions and chromosomes. Although comparative cytogenomics in Brassicaceae essentially explored the collinearity between chromosomes of *A. thaliana* and those of other crucifer species, in the grasses the sequenced genome of *Brachypodium distachyon* served as the source genome for cross-species chromosome painting in *Brachypodium* (Idziak et al. 2011; Betekhtin et al. 2014).

The abundance of dispersed repetitive sequences along plant chromosomes (Schubert et al. 2001) precludes a wider application of BAC-based chromosome painting in comparative plant cytogenomics. BAC-based chromosome painting relies on a BAC library derived from a genome with a low percentage of dispersed repeats and/or their localization is confined to a specific chromosomal region (typically around centromeres). The low repeat abundance decreases the probability that *bona fide* chromosome-specific BAC clones will cross-hybridize to nontarget chromosomes. Thus, BAC FISH and BAC-based chromosome painting was successfully established in plant species with small genome sizes, whose repeat content is low, such as *Amborella trichopoda* (870 Mb), *A. thaliana* (160 Mb), *B. distachyon* (355 Mb), *Genlisea margaretae* (185 Mb), or *Spirodela polyrhiza* (150–165 Mb) (Lysak et al. 2001; Idziak et al. 2011; Chamala et al. 2013; Cao et al. 2016; Tran et al. 2017; Hoang et al. 2018).

#### Oligo Painting

The ever-increasing number and quality of sequenced genomes opened up a new avenue for comparative cytogenomics. Although most plant genomes are dominated by repeat sequences, usually unsuitable for identification of specific chromosome regions, single- and low-copy (coding) sequences are chromosome-specific. The oligo painting approach (Han et al. 2015) is based on designing a library of short synthetic oligonucleotides (e.g., 45–50 bp in length) along megabase-long chromosomes. Such an oligo library is amplified, labeled by haptens or fluorochromes, and single-stranded labeled oligomers (oligo probes) are hybridized to the target chromosomes by FISH (see Jiang [2019] for a recent review). Compared with high-capacity BAC vectors, oligo painting does not require construction of chromosome-specific BAC libraries and subsequent screening to eliminate repeat-rich BAC clones. On the other hand, this methodology requires the availability of a chromosome-level genome sequence, synthesis of high-cost chromosome-specific oligo libraries, and entails several challenging preparatory steps (Han et al. 2015). In addition, oligo libraries offer less flexibility in targeting particular (shorter) chromosome regions compared with chromosome-specific BAC libraries. Oligo painting was successfully used to explore cross-species chromosome collinearity in several model and crop species, such as banana, cucumbers (fig. 2*C* and *D*), maize, or poplar (Han et al. 2015; Filiault et al. 2018; Albert et al. 2019; Šimoníková et al. 2019; Bi et al. 2020; Xin et al. 2020), and its popularity will likely continue to increase. Recently, Bi et al. (2020) modified the original approach by using segment-specific PCR primers that specifically amplify predefined subregions from a single synthetic oligo library (multiplex PCR-based oligo painting, MP-OP; fig. 2C and D). Such double-stranded oligo probes also generate stronger fluorescent signals compared with single-stranded oligomere probes (Han et al. 2015). MP-OP represents a cost- and time-effective approach to pinpoint complex collinearity-corrupting chromosomal rearrangements.

### Comparative Plant Genomics

In the last two decades, the number and quality of sequenced plant genomes have increased sharply, from the first plant genome (*A. thaliana*) sequenced by the Sanger method (The Arabidopsis Genome Initiative 2000), to about 330 sequenced genomes of vascular plants available today (Chen et al. 2019; Kersey 2019). These sequencing projects have enlightened our view on complex patterns underlying chromosomal evolution. Already the pioneering Arabidopsis sequencing project has identified segmental duplications that pointed to a previously not recognized ancient WGD, followed by genome shuffling and descending dysploidy. The constantly improving quality of genome assemblies allows for unprecedentedly detailed comparisons of chromosome structures among closely related species and across phyla. This was achieved particularly through single-molecule sequencing platforms producing longer sequence reads (e.g., PacBio—Pacific Biosciences, Nanopore—Oxford Nanopore Technologies; fig. 2*E*) and by novel methodologies for anchoring contigs and scaffolds into chromosome-scale pseudomolecules (i.e., Hi-C, high-throughput chromosome conformation capture, fig. 2*F*, and optical mapping) (for recent reviews, see Belser et al. 2018; Han et al. 2018; Ho et al. 2020; Michael and VanBuren 2020).

Chromosome-scale genome assemblies provide information on the structure of each chromosome within an organisms’s karyotype, whereas contiguous genome sequences of multiple species allow for a high-resolution detection of intra- and inter-chromosomal rearrangements and structural variations (i.e., sequence variants >50 bp in size, Ho et al. 2020). Aligning genome assemblies of two or more species enables to discover collinearity-corrupting rearrangements and to characterize the corresponding breakpoints with a single-nucleotide accuracy (fig. 2*E*). Thus, sequence-based comparative genomics provides detailed insights into the principles underlying chromosomal evolution. In comparison with comparative cytogenomics, genome sequencing and assembly manifest an increased capability of detecting near-complete spectrum of structural variations, including rearrangements, which were below the detection limit of microscopy-based methodologies, in a time-saving high-throughput manner.

### Comparative Genomics and Cytogenomics

As stated by Hu et al. (2020), “cytogenetics and genomics are commonly used as complementary methods to provide synergistic information regarding chromosome structure.” High-throughput genome sequencing generates data that can be utilized to develop chromosome-specific DNA probes (oligo painting) and novel chromosomal markers, such as tandem repeats (Emadzade et al. 2014; Macas et al. 2015; Báez et al. 2019; Hloušková et al. 2019; Schmidt et al. 2019; Liu et al. 2020). This is a time-saving and cost-effective approach for identifying the most abundant repeats even from low-coverage whole-genome sequence data. Conversely, (comparative) chromosome painting may guide genome assembly (fig. 2*A*–*D*), particularly when a genetic linkage map or reference sequence is lacking. Chromosome-specific probes may help to resolve ambiguities during anchoring sequence contigs and scaffolds to pseudochromosomes. Whole-chromosome comparative cytogenomic maps, such as these based on cross-species hybridization of Arabidopsis BAC contigs, guided genome assembly in several Brassicaceae species (Dassanayake et al. 2011; Willing et al. 2015; Geiser et al. 2016; Nowak et al. 2020), and BAC-FISH navigated genome assembly in a duckweed genome (*S. polyrhiza*; Wang et al. 2014; Hoang et al. 2018). FISH localization of chromosome-specific oligo-paints guided and cross-validated anchoring reference genome sequence to chromosomes in three banana species (*Musa*; Šimoníková et al. 2019).

## Evolutionary Models of Chromosome Number Change

The extensive variation in plant chromosome numbers has been extensively exploited for inferring major genomic events, with particular interest toward determining which species are polyploids and which are diploids. Early work examined the distribution of chromosome numbers within a focal group of species and identified one or more denominators that are common to most chromosome counts. This number, commonly termed *x*, was regarded as the base number and taken to represent the ancestral haploid genome. Consequently, multiplications of this number were treated as the inferred ploidy level of the species. For example, given the following distribution of haploid chromosome numbers {8, 9, 9, 9, 14, 17, 18,18, 18, 20, 27, 27}, nine can be inferred as the base number, species with eight or nine chromosomes may be regarded as diploids, whereas the remaining ones as polyploids. Alternatively, others have designated a species as polyploid if its haploid number was a multiple of the lowest count found in the examined clade by a predefined factor (Stebbins 1938; Wood et al. 2009). Clearly, such threshold methods suffered from extrapolated *ad hoc* assumptions, disregarded the relative frequencies of polyploid and dysploid transitions, and frequently disregarded the phylogenetic relationships among the species.

More recently, chromosome numbers were analyzed within a phylogenetic context following the maximum parsimony principle (Schultheis 2001; Hansen et al. 2006; Ohi-toma et al. 2006). The use of parsimony allows the reconstruction of chromosome numbers at ancestral nodes and the identification of putative transition events along particular branches of the phylogeny. However, as has been well discussed in the literature in the context of molecular sequences, the maximum parsimony approach suffers from several drawbacks (Felenstein 2004). Parsimony does not make use of an explicit model of evolution and thus the same weight is assigned to all state changes: whether they indicate a dysploidy (e.g., 10→11) or a polyploidy (e.g., 10→20) transition, or whether they include one or more transitions (e.g., 10→11, that could occur via a single step, compared with 10→12 that involves at least two transitions). Parsimony ignores branch length information, and thus changes along short branches would be treated similarly as those occurring along very long ones. Similarly, parsimony ignores the possibility of multiple and back transitions occurring along the same branch (e.g., 10→11→10) and thus only provides a lower bound on the number of events that practically occurred. Additionally, the use of parsimony implicitly assumes that the chromosome numbers of extinct ancestral taxa must also be presented in the extant taxa—an assumption that is not necessarily sensible, particularly if rates of chromosome-number change within the group are high.

Over the last decade, methods based on probabilistic models of chromosome number evolution have emerged. These methods are more powerful, as they emulate the evolutionary process along the phylogeny as a stochastic process, while taking into account the mechanisms by which chromosome numbers change. Consequently, the use of such models allows researchers to form and test assumptions regarding the most plausible evolutionary pathways by which the evolution of chromosome numbers have proceeded, while relying on the well-developed machinery of probabilistic statistical inference. For example, the likelihood ratio test can be used to compare the fit of alternative models, each containing a different set of parameters, to a specific data set at hand (Huelsenbeck and Crandall 1997). Additionally, once the evolution of chromosome numbers was casted within a probabilistic framework, a generic modeling scheme was created, allowing modeling extensions to be easily implemented and compared.

Several studies have employed general models of character evolution to model the evolution of chromosome numbers in clades in which chromosome fusion and fission events are the main drivers of karyotype change. For example, Hipp (2007) had employed a series of Brownian Motion and Ornstein–Uhlenbeck processes to examine the evolution of chromosome numbers in the Cyperaceae (sedges), a group characterized by holocentric chromosomes, in which chromosome fusion and fission events are thought to be common and polyploidizations rare (see Dysploidy in Groups with Holocentric Chromosomes above). Rockman and Rowell (2002) have examined the evolution of chromosome numbers in *Planipapillus* (velvet worms), a group characterized by frequent centric fusion events, using the Poisson process. In both these groups, in which the dynamics of chromosomal evolution vary across subclades of the phylogeny, the evolutionary patterns of chromosome numbers better fitted a heterogeneous process. In these studies, chromosome numbers were modeled either as ordered categorical variables or as additive quantitative traits, and thus the possibility of integrating biological phenomena reflecting the mechanisms of chromosome-number change into the models was lacking.

### The *chromEvol* Model

**Fig. 3:**
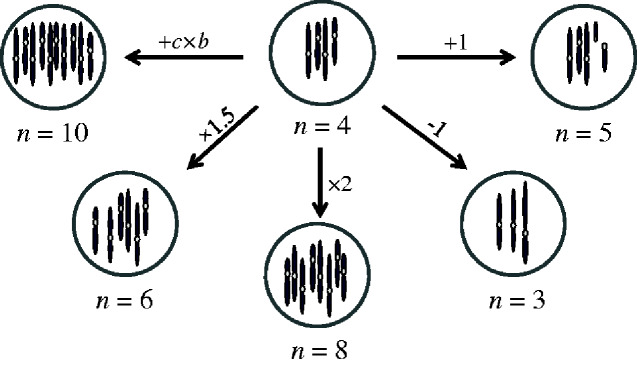
The possible transitions allowed in the *chromEvol* model. The models implemented in *chromEvol* allow for several types of transitions from a genome with *i* haploid chromosomes to a genome with *j* chromosomes: ascending dysploidy (*j *=* i *+* *1), descending dysploidy (*j *=* i *−* *1), genome duplication (*j *=* *2×*i*), demi-ploidy (*j *=* *1.5×*i*), and base-number transitions, in which the addition of any multiplication of a basic number *b* is allowed (*j *=* i *+* c *×* b*; here *c *=* *1 and *b *=* *6).

A model with a specific focus on the evolution of chromosome numbers was first formulated by Mayrose et al. (2010). The *chromEvol* model is based on a continuous time Markov process, which is defined by a rate matrix that describes the instantaneous rate of change from a genome with *i* haploid chromosomes to a genome with *j* haploid chromosomes. The entries in this matrix are determined based on several parameters that define the rate of change for different types of events (fig. 3). The most basic model assumes that three events are possible: WGD (an exact duplication of the number of chromosomes, with rate parameter *ρ*), a single-chromosome increase (ascending dysploidy at rate *λ*), or a single-chromosome decrease (descending dysploidy at rate *δ*). The rate matrix allows for the likelihood function to be computed, given a specified phylogeny and assignment of chromosome numbers to the tip taxa.

The use of the above simple model already allows for several inference tasks: 1) To obtain the maximum likelihood (ML) estimates of the rate parameters, allowing the relative frequencies of the different types of events to be compared; 2) To compare the fit of different model variants, each with different constraints on the free parameters, to a particular data set. For example, one can compare the basic model presented above with three free parameters (*ρ*, *λ*, and *δ*) to a null model, which assumes that polyploidizations are not possible and thus variations in chromosome numbers are only the result of dysploidy events (i.e., *λ*, and *δ* are the two free parameters, constraining *ρ *= 0). Model selection criteria, such as the likelihood ratio test or the Akaike Information Criterion, can then be used to test whether there is a significant evidence for polyploidization in the examined data set; 3) To reconstruct the ancestral chromosome numbers at internal nodes of the tree, including that of the root node. This can be done either using an ML approach (Pupko et al. 2000), in which the single most likely set of ancestral states is inferred, or using a Bayesian approach (Koshi and Goldstein 1996), in which the probability of each chromosome number occurring at each ancestral node is computed; 4) To estimate the expected number of polyploidy and dysploidy transitions that have occurred along each branch of the phylogeny; 5) To assign ploidy levels to extant taxa. A tip taxon can be classified as either diploid or polyploid, with respect to the state at the root of the phylogeny, if the expected number of diploid-to-polyploid transitions from the root to the tip is above (or below) a certain threshold. In initial applications of *chromEvol*, these thresholds were arbitrarily set as fixed values (e.g., 0.9, Mayrose et al. 2011). A more sensitive alternative was developed in Glick and Mayrose (2014), in which a simulation-based approach was used to compute the thresholds that are most suitable to the analyzed data.

We note that a probabilistic model of chromosome-number change was also developed by Hallinan and Lindberg (2011). This model is based on a background birth–death process (allowing for dysploidy transitions) that operates along branches of a species tree combined with the possibility of strict doubling events (i.e., WGD). This model sums over all possible assignments to ancestral states and over all possible number of dysploidy events, while allowing for the possibility of a single polyploidy transition to occur per branch, and can be used to compute the posterior probability that a polyploidy event occurred on each branch of the phylogeny. Extension of this model, to include polyploid transitions aside from exact duplications or multiple polyploidy events, is not trivial since the computation over all possible number of duplications and across all types of transitions per branch need to be explicitly formulated. In the following sections, we thus describe modeling extensions that were developed in the context of the more general *chromEvol* probabilistic framework.

### Variations of the *chromEvol* Model

#### Polyploidy Transitions Other Than Exact Duplications

The basic *chromEvol* model incorporates WGDs that involve exact duplications of the chromosome number. However, polyploid transitions also involve the fusion of gametes with different ploidies. Two types of transitions were incorporated into model variants that allow for such possibilities. In the first, “demi-polyploidy,” occurring at rate *μ*, permits multiplications of the number of chromosomes by 1.5 (Mayrose et al. 2010). This allows, for example, the generation of a hexaploid from a tetraploid lineage via the fusion of reduced and unreduced gametes, or from a diploid lineage in a two-step process via a triploid bridge followed by genome duplication. Note that demi-polyploidy transitions are well defined only for even haploid numbers, whereas for odd numbers, the transition rate is split between the two alternative possibilities. For example, a demi-polyploidy transition from a genome with *n *=* *9 can either lead to *n *=* *13 or to *n *=* *14, both occurring at rate *μ*/2. In this case, the triplication event would unrealistically entail a dysploidy event (either 9→13→26→27 or 9→14→28→27). Furthermore, this modeling scheme is inadequate for some polyploid transitions that involve the combination of genomes with high ploidy levels. For example, in a polyploid series (*n *=* *9, 18, 27, 36, 45), such as that in *Chrysanthemum* (Liu et al. 2012), intercytotype matings that result in 18→45 or 27→36 transitions could not be obtained solely by any combination of demi-polyploidy and WGD events and would erroneously predict some additional dysploidy events (e.g., 18→27→54→53→52…→45).

To overcome these shortcomings, a more general approach was introduced by Glick and Mayrose (2014) and is particularly beneficial for the analysis of clades that exhibit a wide range of ploidy levels, such as the plant genera *Festuca* (*n *=* *7, 14, 21, 28, 35) or *Achillea* (*n *=* *9, 18, 27, 36), whose chromosome numbers are linked by a common denominator that often represents the base number of the group. By incorporating two free parameters: *β*, the base number and *ν*, its respective transition rate, this model allows for any multiplication of an inferred base number to be added to the genome (fig. 3). For example, if *β  = * 9, the transitions from a genome with *n *=* *9 chromosomes to *n *=* *18, *n *=* *27, and *n *=* *36 are allowed in a single step. Notably, this modeling scheme also comes with some shortcomings of its own. For example, it assumes that a clade is defined by a single base number. However, it is possible that due to dysploidy transitions, each subclade in an analyzed phylogeny would be characterized by its own base number or that some subclades would exhibit multiple base numbers. This is in contrast to demi-polyploidy transitions that explicitly account for the current chromosome number of a lineage. Thus, in clades where dysploidy transitions are common, it is conceivable that models that incorporate demi-polyploidy transitions would be better supported than those that include only transitions by an inferred base number. However, because the transitions allowed by the two modeling approaches do not entirely overlap, it is possible that in large clades, in which a large number of parameters could be supported, the inclusion of both transition types would be beneficial.

#### Dependency of the Transition Rates on the Current Number of Chromosomes

The basic *chromEvol* model assumes that dysploidy and polyploidy transitions occur at rates that are identical for all lineages. This implicitly assumes, for example, that a descending dysploidy transition occurring in an *n *=* *20 lineage is equally likely as that of *n *=* *10, whereas in reality, fusion events may be more likely to occur in genomes with large numbers of chromosomes and, accordingly, that the transition rates are related to the number of chromosomes in a lineage. Although this possibility was not yet incorporated for modeling polyploidy transitions, several possibilities were suggested for dysploidy transitions. For example, as implemented in the model by Hallinan and Lindberg (2011), dysploidy rates are forced to linearly depend on the current number of chromosomes (i.e., that the ascending dysploidy rate is *λi*, where *i* is the number of chromosomes in the genome and *λ* is the ascending dysploidy rate). An alternative implementation, incorporated within *chromEvol*, allows this dependency to be tuned using additional free parameters: constant rate parameters, *λ* and *μ* (for ascending and descending dysploidy, respectively), and rate modifier parameters, $\lambda_{l}$ and $\mu_{l}$, that describes the extent of linear dependency. In this case, for example, the ascending dysploidy rate in lineages with *i* chromosomes is $\lambda +\lambda_{l}(i-1)$. A different representation, which allows dysploidy rates to vary exponentially, rather than linearly, as a function of the current chromosome number, was suggested by Freyman and Höhna (2018). Although these options allow for more flexibility in modeling dysploidy transitions, in an analysis of 100 plant genera models that incorporated linear dependency of dysploidy rates on the current number of chromosomes were chosen in merely 2% of the data sets (Glick and Mayrose 2014). This result suggests that the probability of chromosome fission and fusion events is comparable across genomes with different number of chromosomes. However, it is also possible that the latter analysis suffered from low statistical power since all genera analyzed were fairly small (<100 species). It is thus possible that analyses with larger clades would support the additional model parameters and would allow to determine whether dysploidy rates are more likely to increase in genomes with higher number of chromosomes.

#### Nonhomogeneous Processes

**Fig. 4:**
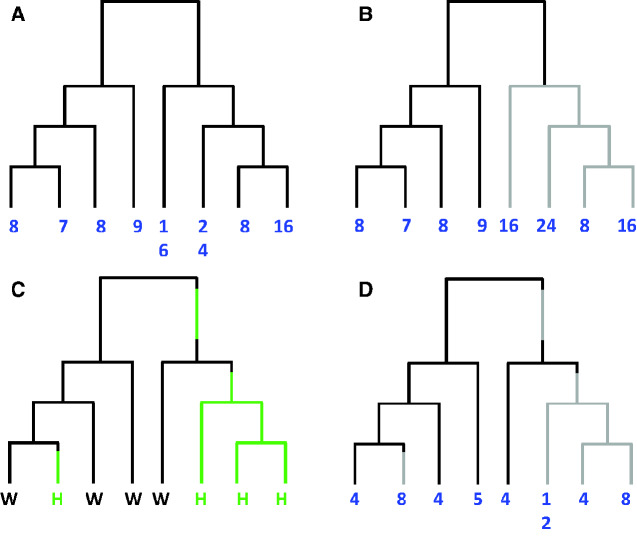
Heterogeneous models of chromosome-number evolution. (*A*) The standard *chromEvol* model assumes that chromosome-number dynamics are similar throughout the phylogeny. In this example, however, the subtree on the left is characterized by low rate of polyploidization and high dysploidy rates, whereas the subtree on the right is representative of a hot spot of polyploidizations. If this clade is analyzed using a single rate matrix (represented by a single color to all branches) the model would not fit the data well and would possibly result in erroneous inferences. (*B*) The use of a split model allows examining whether distinct patterns of chromosome-number change are exhibited in different taxonomic clades. Here, the subtree on the right is a priori classified to have a rate matrix that is distinct from that assumed for the rest of the tree, as represented by gray and black branches, respectively. (*C* and *D*) The effect of a character trait on rates of chromosome-number change. (*C*) A hypothetical mapping describing the evolution of a character trait (here, growth form). W and H denote woody and herbaceous states, respectively, whereas the black and green branches represent the corresponding times spent in each state. In (*D*), this mapping induces distinct patterns of chromosome-number change, represented by black and gray lines, respectively. This could capture a scenario where the polyploidization rate of herbaceous lineages is higher than that of woody lineages and could be modeled using a joint model for the evolution of both chromosome numbers and discrete character traits. Numbers at the tips represent chromosome-number assignments.

The *chromEvol* models detailed thus far assume that the transition pattern is identical throughout the phylogeny. This time-homogeneity assumption is rather unlikely, especially when large phylogenies that include several distinct subclades are analyzed. In such cases, a more realistic approach would allow shifts in the transition pattern: either when different dynamics of chromosome number change are dictated by the presence of a certain organismal trait or when different transition patterns characterize different subclades of the phylogeny (fig. 4).

Márquez-Corro et al. (2019) tested for clade-specific shifts in the pattern of chromosome-number evolution in the large plant family Cyperaceae. The authors found three prominent shifts in the transition process: in the species-rich “non-Siderostictae *Carex*” clade, dysploidy transitions were found to be very frequent; the FAEC clade was typified by karyotype stability with very low rates of dysploidy and polyploidy; the C_4_  *Cyperus* clade, nested within the FAEC clade, exhibited high rates of both dysploidy and polyploidy transitions (allowing for both demi-polyploidy and exact duplications). In the remaining phylogeny, the model inferred frequent descending dysploidies, low rates of ascending dysploidy, and additionally allowed for base-number transitions.

Notably, in the analysis of Márquez-Corro et al. (2019), the original phylogeny was artificially pruned into several smaller trees, and likelihood computations were then performed on each one independently. Thus, information that can be obtained from the pruned trees, which could affect the likelihood computation (i.e., probabilities of ancestral states at the base of each pruned tree) was lost. A more general approach would incorporate the possibility of rate heterogeneity across the phylogeny using a “split model” where different parts of the phylogeny evolve according to different transition patterns of chromosome-number change. Though not yet implemented in any existing modeling environment, this is certainly an area of future development. In addition, such an implementation will allow to pinpoint subclades whose transition patterns deviate the most from the rest of the phylogeny without the need to a priori assign subclades to unique rate matrices. This may be done in a sequential testing approach, similar to branch-site codon models that aim to identify episodic positive selection along certain lineages (Anisimova and Yang 2007), or methods that identify groups with altered diversification patterns along the tree (Alfaro et al. 2009).

Two studies developed modeling extensions that associate patterns of chromosome-number change with the evolution of a discrete character trait (Zenil-Ferguson et al. 2017; Blackmon et al. 2019). Both of these models were developed using the coevolutionary model of Pagel (1994), such that the state space and the data of tip taxa are represented by the pair <chromosome number, character state>. Under the joint model, each character state induces a unique pattern of chromosome-number change with the respective rate parameters, and thus several free parameters are added to the model. For example, assuming a character trait with two possible states (denoted 0 and 1), and the basic *chromEvol* model that allows for dysploidy and WGD transitions, the joint model includes a total of eight free parameters: $\left(\rho_{0},\lambda_{0},\;\mu_{0}\right)$ and $\left(\rho_{1},\lambda_{1},\;\mu_{1}\right)$ that specify the chromosome-number transition pattern under the two states, and two parameters that indicate the rate of change between the two character states. Hypothesis testing can then be derived by defining reduced models with various constraints on the free parameters. For example, a null model in which $\rho_{0}=\;\rho_{1}$ allows statistical testing of the hypothesis that the polyploidization rate is associated with the examined trait. Using such an approach, Zenil-Ferguson et al. (2017) inferred that in eudicots the frequency of both polyploidy and dysploidy is far more frequent in herbaceous plants compared with woody lineages. Notably, in this modeling extension, the transition matrix is largely expanded, which leads to a substantial increase in computational demands and practically limits analyses to character traits with two states and to clades in which the range of observed chromosome numbers is not large. To allow manageable computing times, Zenil-Ferguson et al. (2017) have reduced the size of the transition matrix by mapping any species with >50 chromosomes to an auxiliary “50+” state and further included two additional parameters to represent transition rates between lineages with “50+” chromosomes under the two trait states. Potentially, this limitation may be overcome by using a joint model that first samples probable histories of the character trait and, conditioned on the sampled history, computes the likelihood of the chromosome-number data using a *chromEvol* transition matrix of standard size, as has been developed in the context of joint genotype–phenotype models (Mayrose and Otto 2011; Levy Karin et al. 2017).

All probabilistic models detailed above assume that chromosome-number changes occur continuously in time at rates that are proportional to the branch lengths of the phylogeny. However, polyploidy and dysploidy transitions could frequently lead to reproductive incompatibilities and thus their occurrence should be coupled in time with speciation events (Coyne and Orr 2004), implying their clustering around branching events of the phylogeny. Zhan et al. (2016) have examined over a large cohort of plant genera whether polyploid transitions are temporally associated with speciation by comparing models that allow polyploid transitions to occur alongside speciation events (cladogenesis transitions) to those that assume that they occur continuously over time within a lineage (anagenesis transitions), providing some support for the former. This study was based on a two-step analysis, whereby the ploidy levels of extant lineages were first inferred using *chromEvol* and then probabilistic state speciation and extinction (SSE) models (Goldberg and Igic 2012) were applied to infer the proportion of ploidy shifts that are cladogenetic or anagenetic. A unified model, termed *ChromoSSE*, that distinguishes between cladogenetic and anagenetic chromosome-number transitions for both polyploid and dysploid events was presented by Freyman and Höhna (2018), and implemented within the RevBayes phylogenetic framework (Höhna et al. 2016). This model directly allows determining whether chromosome-number transitions in a specific clade occur primarily within lineages, primarily around speciation events, or in a combination of both processes. The *ChromoSSE* model further employs a reversible jump Markov chain Monte Carlo technique that simultaneously considers the space of all possible models (i.e., any combination of parameters governing the process of chromosome-number change). This alleviates the need to choose a particular set of parameters or to perform model selection, and has the additional benefit that the final inference inheritably incorporates model uncertainty and is not condition on a single model. Applying this model to five plant groups, clade-specific combinations of cladogenetic and anagenetic processes were observed (Freyman and Höhna 2018). Notably, similar to other SSE methods, the performance of the *ChromoSSE* model heavily relies on the accuracy and completeness of the phylogeny (Rabosky and Goldberg 2015; Beaulieu and O’Meara 2016; Louca and Pennell 2020; Shafir et al. 2020) and, since the model includes a large number of parameters, its inferences are expected to be reliable only when large clades are examined. Still, extensions of such models should be particularly exciting as they would allow testing long-standing hypotheses regarding the relationship between chromosome-number changes and patterns of lineage diversification.

## Future Perspectives

Mechanistic phylogenetic models and comparative genomics offer a powerful combination to infer historical genomic processes. To date, a handful of studies have made use of both approaches to better understand the pathways by which the evolution of chromosome number proceeds (Sousa and Renner 2015; Mandáková et al. 2017). Yet, considering that increasingly sophisticated methodologies are constantly being developed, it is expected that more and more studies will make use of a combined strategy in which computational predictions of karyotype evolution are contrasted with empirical data acquired using comparative (cyto)genomics. Here, we have summarized recent progress and highlighted several possibilities that can be incorporated in future modeling developments. Still, current models are focused on changes in chromosome numbers and thus explore only one aspect of karyotype evolution. Modeling other chromosomal characteristics would allow for finer analyses. For example, the *chromEvol* model could be applied to track the number of chromosome arms (the fundamental chromosome number) rather than simply the number of chromosomes. This has the potential to better differentiate between karyotype changes that are due to polyploidization versus multiple dysploidy events (Souza et al. 2015; Mandáková et al. 2017). Furthermore, the karyotype may be represented by the number of chromosomes belonging to different morphological classes, determined by the centromere position (e.g., metacentric, acrocentric, or telocentric). A probabilistic model over this state space could then be formulated by constructing the basic set of allowed transitions (e.g., a Robertsonian translocation would decrease the number of acrocentric chromosomes by two and increase the number of metacentrics by one). A more detailed representation could distinguish the chromosome arms by denoting each arm with a letter. White et al. (2010) used the latter representation to reconstruct intraspecific phylogenetic networks among multiple chromosomal races of the house mouse and common shrew. Their method is built upon the computation of the distance between any two examined karyotypes (defined by the number of whole-arm reciprocal translocations and Robertsonian fusions and fissions that are required to transform one karyotype to another). The usability of such an approach for among-species analyses is still unclear, but a main limitation of its widespread use is data availability, since detailed karyotype representations are not available for many species. Moreover, this approach requires that homeology relationships among chromosome arms are resolved, which is not trivial when increasingly distant lineages are compared. Another fruitful endeavor should be to model the evolution of chromosome numbers together with other informative genomic attributes. One obvious candidate is genome size, which is expected to increase simultaneously with chromosome number after a polyploidization event, but could take on different trajectories at longer time intervals. For example, genome size itself could influence patterns of chromosome-number change as additional rounds of polyploidizations are less likely to occur in genomes of larger size (Zenil-Ferguson et al. 2016), whereas increased repeat copy numbers theoretically provide more abundant substrates for DSB misrepair underlying dysploid changes. Certainly, such future methodological advancements would allow for refined understanding of how major genomic events, such as dysploidy and polyploidy, have shaped the karyotype of extant and ancestral lineages.
